# Evaluation method of medical service system based on DEMATEL and the information entropy: A case study of hypertension diagnosis and treatment in China

**DOI:** 10.1371/journal.pone.0243832

**Published:** 2020-12-14

**Authors:** Rui Miao, Xiaohao Xiang, Qi Wu, Zhibin Jiang

**Affiliations:** 1 School of Naval Architecture, Ocean & Civil Engineering, Shanghai Jiao Tong University, Shanghai, China; 2 China Institute for Urban Governance, Shanghai Jiao Tong University, Shanghai, China; 3 School of Mechanical Engineering, Shanghai Jiao Tong University, Shanghai, China; 4 Antai College of Economics and Management, Shanghai Jiao Tong University, Shanghai, China; Shandong University of Science and Technology, CHINA

## Abstract

Precise and reasonable evaluation of the multi-attribute value of medical system is the basis for hospitals to implement total quality management. Excellent medical system is necessary as a part of modern urban governance. However, most of medical value evaluation work relies on scale and artificial scoring at present, lacking in objectivity. Therefore, a scientific and comprehensive medical value evaluation system is needed urgently to give full play to the guiding role of value evaluation and promote the improvement of the medical service system. In this study, DEMATEL and information entropy are used to quantify the degree of mutual influence between system indicators and the differences in medical market performance respectively, so as to obtain the objective index weight. Hypertension has the highest incidence in the whole chronic disease system, which seriously affects people's daily life. Based on the existing hypertension diagnosis and treatment index system, a comprehensive and objective evaluation model is established to evaluate the hypertension diagnosis and treatment behaviors of different medical institutions, which achieves good result. This method has effectively improved the relative deficiency of one-sided subjective evaluation and has a great guiding significance for the comparison of treatment in departments and the economical use of medical resources.

## Introduction

There are many problems in Chinese medical service system currently. The insufficient medical resources and low utilization rate as well as partial medical institutions' one-sided emphasis on economic benefits and neglect of patient satisfaction and social justice, all lead to problems such as "difficult and expensive access to medical services" [1–3]. Medical service system is important in the field of urban governance. Successful urban governance is able to provide both various and superior medical service. In order to promote the medical institutions for optimizing the utilization efficiency of medical resources and improve their service level as well as patient satisfaction, it is urgent to establish a set of objective and accurate evaluation mechanism for the overall multi-attribute value of medical institutions [4, 5]. The evaluation method can also enhance the competitive awareness of hospital managers and optimization service awareness [6].

At present, most of medical value evaluation work relies on scale and artificial scoring [7, 8]. How to overcome the subjectivity of artificial scoring and establish a scientific, standard and operable evaluation system has become a significant difficulty in medical value evaluation [9].

This could be solved by the following ways: firstly, select objective and quantifiable evaluation indexes; secondly, adopt the objective evaluation method of the index weight [10–12]. Some scholars have conducted in-depth research on the comprehensive evaluation method of hospital medical value [13]. Chen Wei et al. established a hospital service value-evaluation system through literature analysis and expert consultation, quantified the weight of indicators by using Analytic Hierarchy Process, and established a comprehensive evaluation model by using weighted comprehensive index method, weighted rank-sum ratio method, and weighted TOPSIS method [14]. Wang Zhouqiang et al. introduced the corrected Ridit method combined with RSR method to comprehensively evaluate the medical value of clinical departments, making the evaluation results more objective [15]. Song Weicai et al. introduced the data envelopment analysis method into the evaluation of medical benefits and values of primary hospitals, and proposed measures for increasing efficiency and reducing consumption of primary hospitals [16]. Tian Changjun et al.'s evaluation scale based on patient experience reflects the value of medical services [17]. However, due to the subjectivity of scoring, the scoring results are not the same, and the practical operation is complicated [18, 19].

From the existing literature, the comprehensiveness and representativeness of the selection of evaluation indicators are mostly demonstrated in the process of establishing the medical value evaluation system, but the objective quantitative method of index weight has not been deeply studied [20]. In the process of establishing the multi-attribute evaluation system of medical value, most of studies demonstrate the comprehensiveness and representativeness of index selection but do not conduct in-depth studies on the interaction between various index variables [21, 22]. Different medical indexes often influence each other in the process of medical service system providing health services to patients, and the improvement of one evaluation index may affect the performance of others [23]. For instance, enhancing the level of medical staff and the use of the best drugs can have a positive effect on patient satisfaction, hospital social reputation and other indicators. However, it pushes up the medical cost, thus costs per patient treated will inevitably increase. As a result of such interaction relations, a value indicator with a relatively low degree of importance should receive higher attention, for the improvement of this index will lead to the improvement of other health value indicators. Therefore, it is necessary to establish a set of feasible and straightforward medical value evaluation model which reflects the internal relations of indicators and serves as the assessment standard for hospitals to improve service value [24].

According to the statement above, in order to be close to the reality, this study needs to make a scientific evaluation of the correlation between the competitive evaluation results of medical service institutions and the medical health value index, so as to correct the deficiency of subjective weight. The Decision-Making and Trial Evaluation Laboratory (DEMATEL) can not only express the mutual influence relationship between each value index but also quantify the size of the mutual influence relationship between each index [25–27]. Therefore, this method can be used to measure the mutual influence relationship between each index of medical health value. In the meantime, the current development and construction of medical institutions rely on medical technology excessively, however the medical treatment process, scientific management, the development of medical service specialization and differentiation are ignored. All of the improper actions result in serious homogenization competition among medical service institutions because they cannot meet the medical needs of patients at different levels [28]. Then the patient satisfaction is generally low. The influence of the competitive differentiation evaluation results of medical service institutions on the importance of value indicators by using the information entropy method can promote the development of differentiated competition among medical service institutions, help medical institutions form their characteristics and meet the diversified medical needs of patients [29, 30].

### Ethics statement

The research was reviewed and approved by the National Natural Science Foundation of China (No. 71432006) and the Ethics Committee of Shanghai Jiao Tong University (No.S-1281/2016). Written consent was obtained.

## Value evaluation theory and method

### Construction of the value index system

There are many comprehensive evaluation indexes of medical service value, but the medical value evaluation indexes currently used by different hospitals in different regions vary considerably. There are problems such as the one-sided emphasis on treatment effect, neglect of essential quality and process quality evaluation, and lack of comprehensive evaluation including economic benefits [31, 32]. In the process of constructing the index system of medical service value, considering that the participants of medical service activities composed of patients, hospitals and governments have different value orientations, the indicators should be systematic and the benefits obtained by different subjects should be comprehensively considered. At the same time, in order to ensure the evaluation results’ veracity and objectivity, the indicators must be quantifiable [33].

On the basis of reviewing and summarizing relevant research literature, interviews with experts, and different medical evaluation systems, this study obtains the value of medical service system by measuring the input and output of medical resources in the system. The input part mainly includes government medical resource input and social resource input, and output part includes not only the improvement of patient health benefit, but also the economic benefit brought by medical behaviors and other social benefit [34].

Health benefit, as the most fundamental and important output of medical service, reflects the growth of the health utility of patients after receiving medical service [35]. Social benefit is embodied in the social and public welfare of medical behaviors, such as the utility of social harmony brought about by the improvement of patient satisfaction, the promotion of social equity and the progress in social medical standards [36]. Economic benefit is mainly embodied in the driving effect of the medical economy and industrial development, which can be measured from profitability, growth, and productivity [37]. Resource consumption represents all resources consumed to provide medical service [38].

Therefore, this study measures the overall input of medical resources in the system from the resource consumption dimension, and measures the output of medical services in the system from the three dimensions of health benefit, economic benefit, and social benefit. This study divides the evaluation index system into two levels, the dimension level and the index level. The dimension level includes three output dimensions (health benefit, economic benefit, social benefit) and an input dimension (resource consumption). In the evaluation of medical service system, comprehensive quantification of each dimension is the focus of value analysis, so the choice of index level is very important.

No matter what kind of medical service mode is, the ultimate goal pursued by patients and doctors is the health benefit(dimension A). It is also the most important output of the medical system. A comprehensive evaluation of life and health effects should be centered on patients and geared to the needs of process, structure, and result. The existing evaluation system pays little attention to the essential quality and process quality while this study considers the essential quality, link quality and final quality from three aspects, combine them with the existing index system and chooses five health benefit indexes such as doctor-patient ratio(A1), the proportion of senior title of professional doctors (A2), annual outpatient number(A3), diagnostic coincidence rate (A4) and cure rate (A5).

According to the subject, the resource consumption (dimension B) of the medical system can be divided into direct resource consumption and indirect resource consumption caused by diagnosis and treatment. However, according to the types of resource consumption, it can be divided into medical resource consumption and social resource consumption. The consumption of medical resources can be measured by the cost of drugs(B1), cost of equipment and beds(B2), cost of medical personnel(B3) and other indicators, while the consumption of social resources includes the medical insurance funds invested by the government(B4). Under the current situation within limited medical resources, reasonable allocation of the above medical resources is the key to improve the value of the medical system.

As a significant part of public social services, medical and health services should emphasize their social benefit (dimension C) and sociality and public welfare(C3) is an important aspect. From the perspective of public welfare of medical resources, medical insurance and medical institutions have inescapable social responsibilities in supporting medical treatment for poor patients and social public medical services. The sociality of diagnosis and treatment requires medical institutions to strive to improve patient satisfaction(C1) and scientific research output(C2), and assume their due social responsibilities.

The economic benefit (dimension D) of medical services mainly include three aspects: profitability, growth, and productivity. The profitability of medical institutions is reflected in the pulling effect of the medical industry on social economy, which mainly includes three indicators: equipment bed income(D1), average out-patient cost per time(D2) and average drug cost per time(D3). Growth means that the profitability of medical institutions needs to be able to support their development, which can be measured by the cost-return index(D4). The productivity of doctors, however, is mainly reflected in the per capita business income of doctors(D5), which reflects the economic benefit of hospitals.

In summary, the four first-level dimensions can be divided into 17 sub-indexes, which are the diversification and further expansion of each dimension respectively. The detailed indexes are shown in Table 1.

**Table 1:** The first-level dimensions and sub-indexes.

First-level Dimensions	Sub-Indexes
health benefit **A**	doctor-patient ratio(A1), the proportion of senior title of professional doctors(A2), annual outpatient number(A3), diagnostic coincidence rate (A4), cure rate (A5)
resource consumption **B**	drug costs per patient (B1), the cost of equipment beds per patient (B2), medical personnel cost per patient(B3), insurance subsidies per patient (B4)
social benefit **C**	patient satisfaction (C1), research output rating (C2), public welfare medical support (C3)
economic benefit **D**	equipment-bed cost per patient (D1), second treatment costs (D2), second drug cost(D3), cost yields(D4), doctors’ business incomes per head (D5)

### Methods for measuring index weight

Because of the correlation between the indicators of medical service system, the importance obtained by AHP and other methods may not be accurate. DEMATEL method is proposed to correct this defect quantitatively. DEMATEL method can not only express the mutual influence relationship between each value index but also quantify the size of the mutual influence relationship between each index. Therefore, this method can be used to measure the mutual influence relationship between each index of medical health value [39].

Besides we can conclude the advantages and disadvantages of different medical institutions and find out what needs improving through the horizontal comparison of the performance of various indicators of medical institutions in the medical market. When the evaluation value of a specific index is same, we can assume that different hospitals have substantial homogeneity and consistent performance in this index. The improvement of this index can meet the diversified service needs of patients and promote differentiated competition of different medical institutions in different aspects. Therefore, the system should attach higher importance to this factor index. According to the transverse comparison results in the medical service market, we can give different weights for indexes and guide the development of the medical service system of differentiation competition, which shares the same idea with information entropy theory precisely [40]. Therefore, this study introduces the method of information entropy to deal with competitiveness evaluation information, obtain the correction importance of index and make the index weight more objective. This evaluation method adding information entropy can promote the development of differentiated competition among medical service institutions, help medical institutions form their characteristics and meet the diversified medical needs of patients.

In summary, methods of DEMATEL and information entropy for measuring index weight have been formed. Firstly, use DEMATEL method to analyze the degree of the correlation between the indexes. Secondly, use the composite-influence matrix T to modify the importance of indexes. Thirdly, analyze the competitiveness evaluation based on the concept of information entropy to obtain the correction of the importance of indexes. Finally obtain the ultimate importance of indexes. The detailed steps are as follows:

1. According to the expert investigation, AHP is used to obtain the fundamental importance ratings of n medical value indexes of *V*_1_,⋯,*V*_*n*_ indicators in *C*_1_,⋯,*C*_*k*_ hospitals. The ratings are $w_{0}^{}=(w_{0}^{1},\cdots ,w_{0}^{n})$.
2. The influence matrix A is built based on DEMATEL theory. The correlation strength between the indicators is defined as 0–4, representing the relationship from no correlation (0) to strong correlation (4). The correlation strength of index i to index j is obtained by scoring by experts denoted as *a*_*ij*_. The correlation relation between all indexes is expressed by the matrix and the direct influence matrix A is obtained. Then, the order of the direct influence matrix with N evaluation indexes is order N by N. The influence matrix A is shown as follows.
  $$A=\left[\begin{array}{cccc} a_{11} & a_{12} & \ldots & a_{1n}\\ a_{21} & a_{22} & \ldots & a_{2n}\\ \ldots & \ldots & \ldots & \ldots \\ a_{n1} & a_{n2} & \ldots & a_{nn} \end{array}\right]$$ (1)
3. Normalize direct-influence matrix M. In order to quantify the indirect influence relationship between the two factors, the above matrix A needs to be normalized and the normalized influence matrix D is obtained. By adding the rows and columns of the direct influence matrix A, we can obtain the total direct influence of factor i on other index factors. $\underset{1\leq i\leq n}{\max}\sum_{j=1}^{n}a_{ij}$ represents the total influence degree of the factors that have the most significant influence on other factors while $\underset{1\leq i\leq n}{\max}\sum_{j=1}^{n}a_{ij}$ represents the total direct influence degree of the factor index that is influenced most. The positive value M takes the maximum value between the two. The normalization method is to take the maximum value M of the sum of the elements of each row (or column) in the matrix, and then the matrix A divided by M to get the normalized influence matrix and get the elements in the matrix D between 0 and 1. Matrix D and M can be obtained as follows:
  $$\begin{array}{l} M=\max\left(\max\sum_{j=1}^{n}a_{ij},\max\sum_{i=1}^{n}a_{ij}\right)\\ D=A\times \frac{1}{M} \end{array}$$ (2)
4. Calculate the composite-influence matrix T.
  $$T=\underset{m\to \infty }{\lim}(D+D^{2}+\cdots +D^{m})=\sum_{m=1}^{\infty }D^{i}$$ (3)
  T represents the sum of m-order comprehensive influence between the indicators. *t*_*ij*_ represents the comprehensive influence of i exerts on j. As $\sum_{m=1}^{\infty }D^{i}=D\left(I+D^{1}+\ldots +D^{m-1}\right)=D{\left(I-D\right)}^{-1}\left(I-D^{m}\right)$, we have the composite-influence matrix T presented as follows:
  $$T=D{\left(I-D\right)}^{-1}=T{\left(t_{ij}\right)}_{n\times n}$$ (4)
5. Use *T*(*t*_*ij*_)_*n*×*n*_ as the fundamental importance of the cross support matrix to correct the index factors. This is denoted as the correlation importance as shown in follows:
  $$w_{T}^{i}=\frac{\sum_{j=1}^{n}t_{ij}w_{0}^{j}}{\sum_{i=1}^{n}\sum_{j=1}^{n}t_{ij}w_{0}^{j}}$$ (5)
6. Evaluate the performance of *V*_*j*_ in k hospitals and get the results *r*_*i*1_,⋯,*r*_*in*_. Let $r_{i}=\sum_{j=1}^{n}r_{ij}$, and then the series $p_{ij}=\frac{r_{ij}}{r_{i}}$ can be regarded as the sample of *V*_*i*_ in k hospitals which obeys the discrete distribution. The formula of information entropy quantization for *V*_*i*_ is shown in follows:
  $$E\left(p_{i1},\cdots ,p_{in}\right)=-\varphi_{n}\sum_{j=1}^{n}p_{ij}\ln\left(p_{ij}\right)=-\varphi_{n}\sum_{j=1}^{n}\frac{r_{ij}}{r_{i}}\ln\left(\frac{r_{ij}}{r_{i}}\right)$$ (6)
  Generally, we let $\varphi_{n}=\frac{1}{\ln\left(n\right)}$ to ensure 0≤*E*(*p*_*i*1_,⋯,*p*_*in*_)≤1.
  Information entropy *E*(*V*_*i*_), *i*∈(1,*m*) is geometrically mapped into the interval (0,1) and denoted as competitive importance $w_{S}^{i}$ to reflect the differences between the existing hospitals. The $w_{S}^{i}$ is presented in follows:
  $$w_{S}^{i}=\frac{E\left(V_{i}\right)}{\sum_{i=1}^{m}E\left(V_{i}\right)}$$ (7)
7. On the basis of fundamental importance for each index, we obtain the final importance *f*_*i*_ through a weighted average of competitive importance $w_{S}^{i}$ and correlation importance $w_{T}^{i}$. *f*_*i*_ is shown as follows:
  $$f_{i}=\alpha w_{T}^{i}+\left(1-\alpha \right)w_{S}^{i}$$ (8)

The value of the proportionality factor *α* reflects the tradeoff between the relative importance which considers the mutual influence and the competitive importance which reflects the horizontal evaluation results among the medical service organizations in determining the final importance of the health value indicator factors.

There are four dimensions of indicators in the medical service value evaluation system. Each index has different dimensions and types, so it cannot be calculated and analyzed but standardized. Find the maximum value of each variable in the data matrix, and divide the original data of each variable by the maximum value, so that we can obtain the normalized data. This method can map all the original data to 0–1 and ensure the transformed data are non-dimensional, which is convenient for comparison. In the meanwhile, it maintains the linear relationship between the original data values. After the index of life and health benefit, economic benefit and public welfare effect are standardized and the value of each index is converted into 0 to 1, the comprehensive calculation can be carried out.

In traditional medical value research, *F*_*i*_ of medical value can be obtained by simply weighting and summing. The formula is as follows:

$$F_{i}=\sum_{j=1}^{n}f_{j}z_{ji}$$ (9)

The purpose of this study is to evaluate the value of medical services from a social perspective and guide the rational allocation of medical resources. Porter defined the value of medical services as the ratio between effect and cost and believed that only with the goal of improving the value of medical services can stakeholders of medical services (such as hospitals, doctors, nurses, patients, etc.) be united to improve the effect of medical services. The value thus defined includes the concept of efficiency. In the medical service evaluation system of this paper, the medical service effect includes three dimensions: health benefit(L), social benefit(S) and economic benefit (E). The cost of medical behavior is mainly reflected in the consumption of medical resources(C). Therefore, the quantitative medical service value evaluation formula can be written as:

$$V_{i}=\frac{\alpha L_{i}+\beta S_{i}+\theta E_{i}}{kC_{i}}$$ (10)

## Case study

The incidence of chronic diseases in China presents a gradually increasing trend. Senior citizens have become the main patients of various chronic diseases due to the decline in the function of the body organ system, the cardiopulmonary reserve, nutrient metabolism, and immunity. By the end of 2017, the prevalence of chronic diseases in people over 65 years old in China was 64.5 percent. Among them, hypertension has the highest incidence in the whole chronic disease system, which seriously affects people's daily life. In addition, this kind of disease has a long course, which requires regular monitoring of disease development and timely intervention to prevent dangerous situations [41–42]. Therefore, how to scientifically and effectively control chronic diseases represented by hypertension has become a key and difficult problem in clinical research. Currently, high-level hospitals, community health institutions, and mobile medical platform share responsibility for regular monitoring on patients with high blood pressure. The three types of medical institutions have different characteristics: the high-level hospital has fully equipped outpatient services and high medical level, but cannot meet the demand of all patients hospitalized. Regular patients queuing to register need to wait for a few hours or even a few months; Community health institutions can provide residents with necessary, low-cost, nearby and fast medical and health services; the Mobile medical platform has excellent advantages in dynamic monitoring and service accessibility, but its medical level is relatively weak.

The index weight measurement of hypertension diagnosis and treatment can be finished by using DEMATEL and information entropy method. Most patients with stable hypertension should first be recommended to the medical institutions with the highest overall score for regular examination and treatment to optimize the efficiency of medical resource utilization and save treatment costs for patients.

In this paper, the specific performance of hypertension patients in departments of A, B and C hospitals in a particular region of Shanghai was compared and analyzed. Among them, A is a high-level hospital, B is a community hospital, and C is a mobile diagnosis and treatment platform.

### Data collection

The initial weight data of indexes and scores of these indexes belonging to the three medical institutions are obtained through the medical management department’s statistics cooperation with the national natural science foundation of China and a sample survey of scores from patients and senior experts in the field of hypertension. In the process of sample collection, a total of 108 hypertensive patients in Shanghai with a medical history of more than one year were issued patient questionnaires and 35 senior experts in the field of hypertension were issued expert questionnaires. Because some of the questionnaires are closely related to these three types of medical institutions, it is necessary to eliminate questionnaires from patients and experts who do not fully understand these three types of medical institutions. Besides questionnaires were selected by observing the vacancy rate and continuous rate of the questionnaire responses. After screening, the valid questionnaires were 92 patient questionnaires and 30 expert questionnaires. After the actual measurement of indicators and the standardized data processing, the standardized evaluation scores of 17 sub-indexes in the three selected hospitals for the treatment of hypertension are shown in Tables 2–5.

**Table 2:** Health benefit Ⅰ the standard value of sub-indexes.

Institution	Health Benefit A				
	*A*_*1*_	*A*_*2*_	*A*_*3*_	*A*_*4*_	*A*_*5*_
**A**	0.300	1.000	1.000	1.000	1.000
**B**	1.000	0.660	0.250	0.908	0.959
**C**	0.200	0.205	0.063	0.816	0.804

**Table 3:** Resource consumption Ⅱ the standard value of sub-indexes.

Institution	Resource Consumption B			
	*B*_*1*_	*B*_*2*_	*B*_*3*_	*B*_*4*_
**A**	1.000	1.000	1.000	1.000
**B**	0.604	0.667	0.642	0.700
**C**	0.792	0.333	0.100	0.100

**Table 4:** Social benefit Ⅲ the standard value of sub-indexes.

Institution	Social Benefit C		
	*C*_*1*_	*C*_*2*_	*C*_*3*_
**A**	0.867	1.000	0.857
**B**	1.000	0.600	1.000
**C**	0.746	0.100	0.286

**Table 5:** Economy benefit Ⅳ the standard value of sub-indexes.

Institution	Economy Benefit D				
	*D*_*1*_	*D*_*2*_	*D*_*3*_	*D*_*4*_	*D*_*5*_
**A**	1.000	1.000	1.000	1.000	1.000
**B**	0.518	0.600	0.492	0.233	0.250
**C**	0.120	0.200	0.714	0.488	0.063

### Measurement of index weight

**Fig 1:**
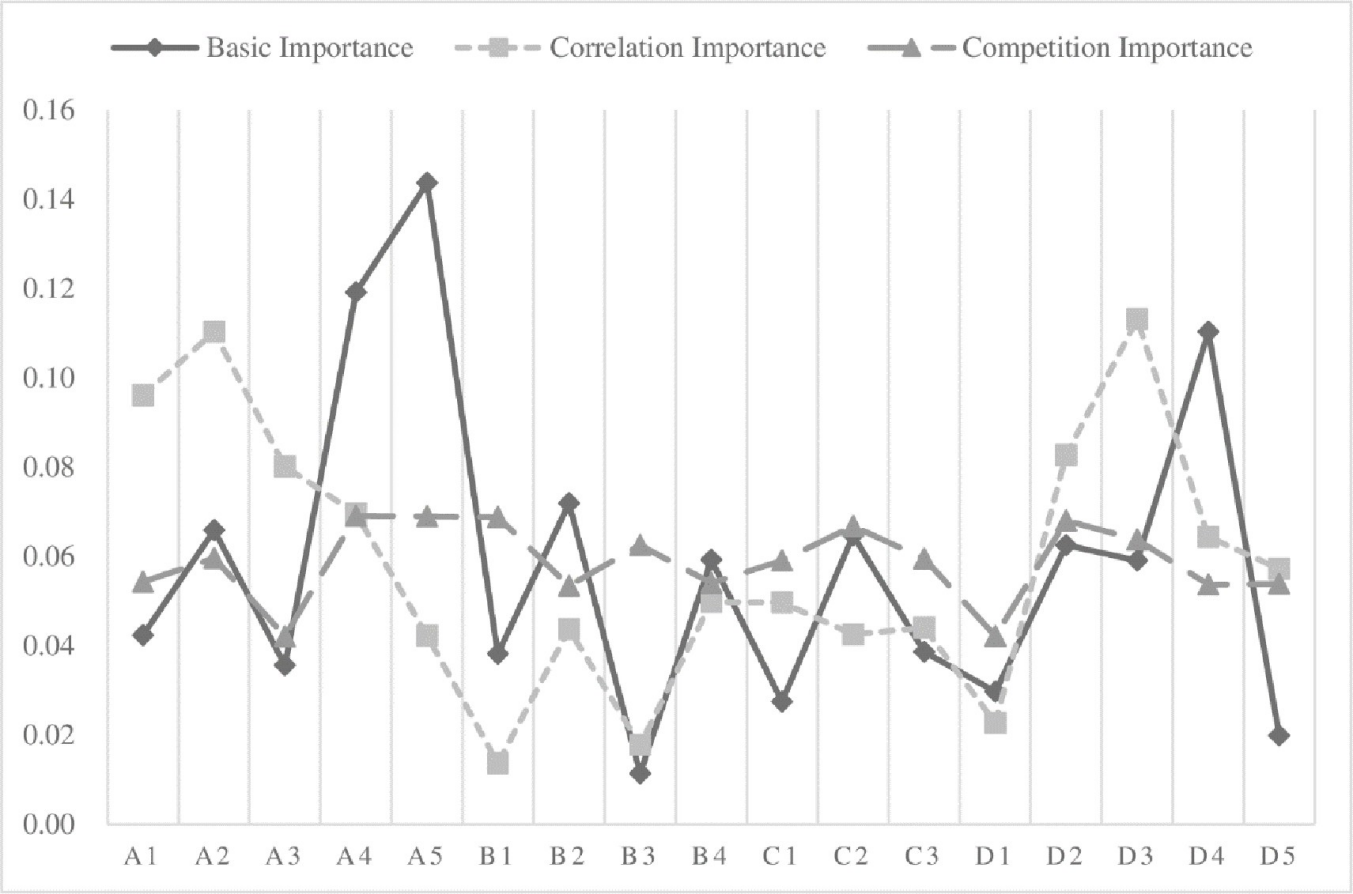
Numerical variation chart of basic importance, correlation importance, and market importance.

According to step 1(section 2.2), the basic importance of sub-indexes is shown in Fig 1 (blue line). Among them, health benefit dimension has the largest weight, with a total weight of 0.407. Health benefit is always the main goal in the medical service system.

DEMATEL method is used to analyze the degree of interaction between the sub-indexes. According to step 2-5(section 2.2), the rank of correlation importance of 17 sub-indexes influencing each other is shown in Fig 1 (orange line). It can be seen that after considering the mutual influence of index, the importance of each index changes to different degrees. After considering the mutual influence, the trend of the correlation significance curve is roughly the same as that of the essential significance, indicating that although the correlation significance changes, it can still reflect the primary distribution of the index weight before the adjustment.

**Fig 2:**
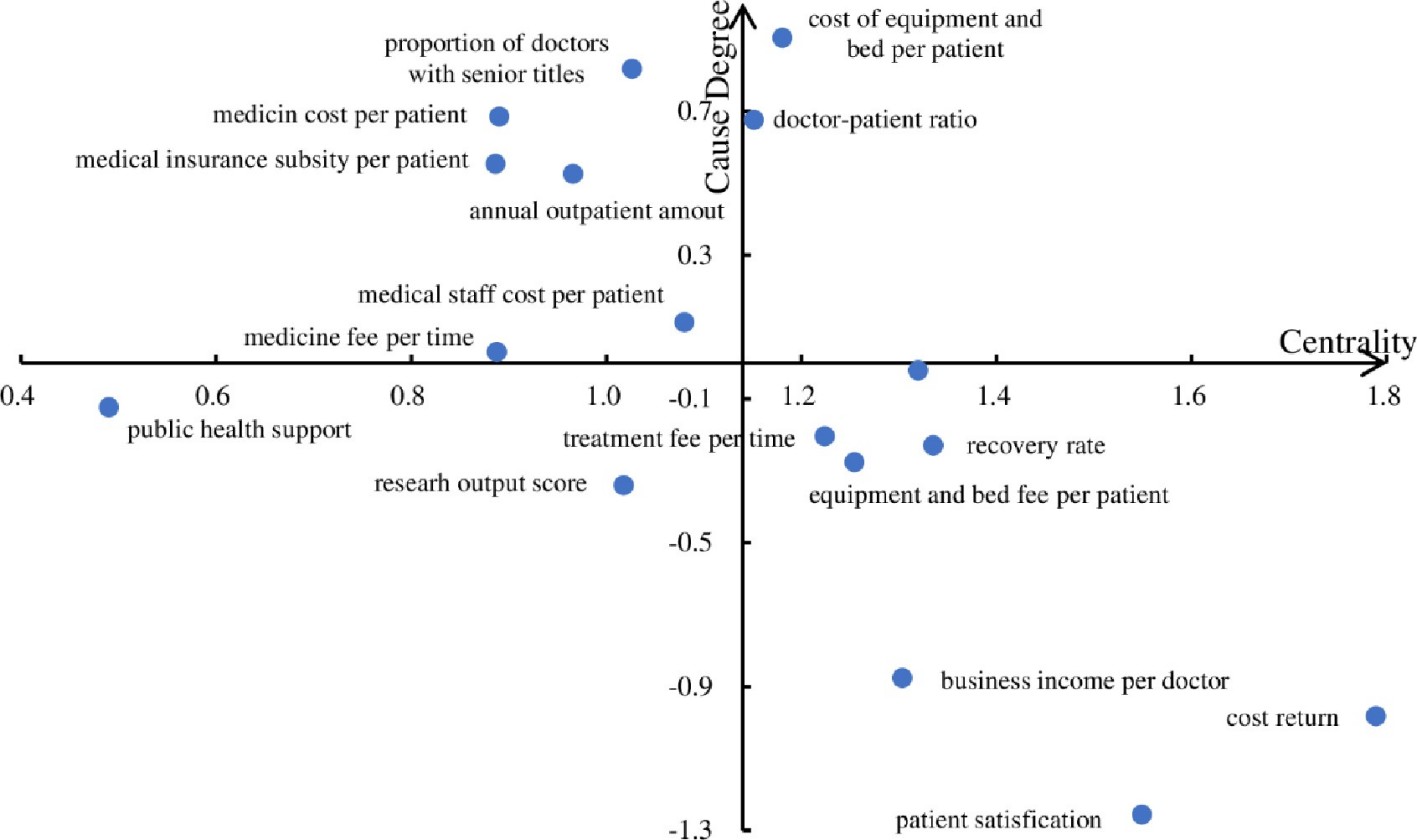
Causal influence diagram among the three indicators.

The row and column of the composite influence matrix T are respectively added to obtain the corresponding influence degree *r*_*i*_ and the influence degree *c*_*i*_. The center degree (*r*_*i*_+*c*_*i*_) and the cause degree (*r*_*i*_−*c*_*i*_) are used as the horizontal and vertical coordinates to draw the causal influence diagram, as shown in Fig 2. Centrality reflects the role of indicators in the evaluation system. Among them, indicators such as cost rate of return (D4) and patient satisfaction (C1) have the highest centrality, which indicates that many links will have an impact on these indicators. Indicators with positive cause degree, such as equipment bed cost per patient(B2) and doctor-patient ratio (A1), indicate that they have a high impact on other indicators. The improvement of the evaluation value of such indicators can have a positive impact on the performance of other indicators, and the weight increases after the DEMATEL method is used. As for indicators belonging to outcome factors, such as patient satisfaction (C1), although a high centrality value indicates that it plays a significant role in the evaluation system, a negative causality degree with a considerable absolute value indicates that it is greatly affected by other factors and its improvement should be started from the causality index that affects them. So that the weight of those outcome indicators is reduced. There is another category of indicators, such as diagnostic coincidence rate (A4) and average drug cost per time (D3) whose reason degree is close to 0. This indicates that such indicators have little impact on other indicators and are relatively independent. The above changes are beneficial to the process factors that the assessed system attaches importance to value enhancement and the results obtained by using the model are also in line with expectations.

Eqs (5) and (6) quantify the information entropy of the evaluation values of each index of the three medical institutions to reflect the degree of difference of the performance of each index in the current medical service system. They also normalize the significance of competition, as shown in Fig 1 (gray line). On the whole, there is little difference in the significance of competition among various indexes, which indicates that the performance of various medical institutions is relatively consistent. The clinical coincidence rate (A4), the cure success rate (A5) and the cost-related index competition are of great importance. Improving the performance of such indicators is conducive to effectively promoting the differentiated competition and character development among medical institutions, so they should be given higher weights.

### Comprehensive evaluation result

According to the steps(section 2.2), based on the relative importance of mutual influence relations and competition importance of information entropy, the weight of four dimensions has been obtained as shown in Table 6. The synthesis weights are obtained according to the Eq (8) with respectively 70% and 30% and the Eq (9). The result shows that health benefit, resource consumption, economic benefit, and social dimension weight decreased in turn.

**Table 6:** The weight distribution of each dimension.

	Health Benefit A	Resource Consumption B	Social Benefit C	Economy Benefit D
**Basic Importance**	0.407	0.252	0.122	0.22
**Correlation Importance**	0.399	0.317	0.075	0.209
**Competition Importance**	0.294	0.239	0.185	0.282
**Comprehensive Weight**	0.367	0.294	0.108	0.231

**Fig 3:**
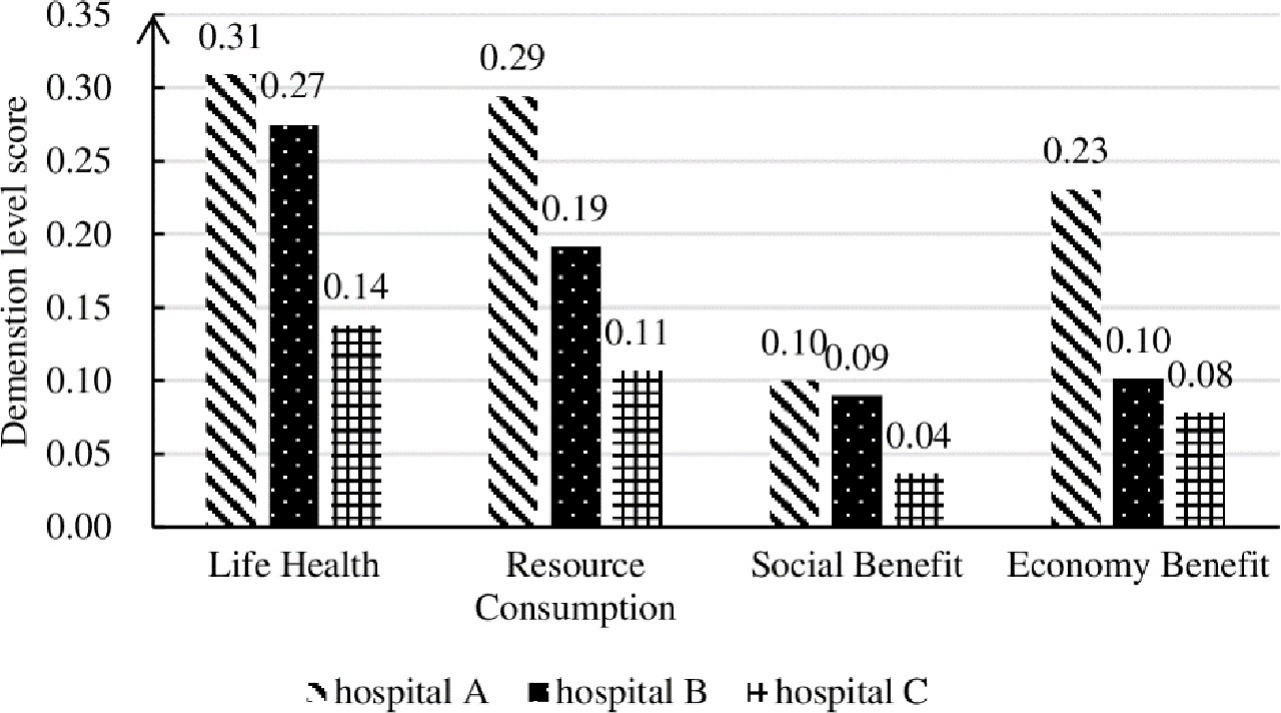
The weighted scores of each dimension in the three hospitals.

Fig 3 shows the four-dimensional scores of A, B and C institutions based on the sub-indexes and the final weighting. As for health benefit A, social benefit C and economic benefit D, the higher the score, the better for the institution. While for the dimension resource consumption B, the lower scores represent the less consumption of health care and social resources. According to the evaluation results, the value of benefits evaluation of each dimension generated by hospital A, B and C decreases in turn, and the value of resource consumption also decreases in turn. The evaluation results are also in line with the actual operation of the hospital. When using Eq (10) to compare the service value of the medical system in terms of effect-cost, we can find that the higher the value of output benefit evaluation and the lower the value of resource consumption, the higher the medical value will be. The hospital medical value evaluation results of A, B, C are in the order: 2.1731, 2.4295, 2.3615. The hospital B is optimal and then C and A in turn. This result indicates that in the treatment of hypertension, although high-level hospital A produces more benefits in dimensions such as healthy living, the community hospital B still gets a higher score due to its relatively small resource consumption. The score of mobile diagnosis and treatment platform C in the dimension of life and health is significantly lower than that of hospital A and B which means it needs to improve its level in several aspects of medical technology and medical conditions.

## Discussion

In this study, using DEMATEL and information entropy method and combining with Porter cost-effectiveness medical evaluation model, we can obtain a comprehensive, objective evaluation model to evaluate medical service value. DEMATEL and information entropy method are effective in the weight evaluation of medical value indicators, which minimize the impact of direct artificial weighting on the evaluation results and ensure the evaluation results' objectivity. DEMATEL's method objectively quantifies the mutual influence between the indicators while information entropy method is used to measure the degree of differentiation of competition among medical service institutions and to increase the index weight of homogenization of current medical institutions. The use of information entropy can promote the development of differentiated competition among medical service institutions and help medical institutions form their characteristics and meet the diversified medical needs of patients.

The model is applied in the case where hypertension diagnosis and treatment behaviors of three medical institutions, namely, high-level hospital A, community hospital B, and mobile diagnosis and treatment platform C are evaluated. From the results that are basically in line with the current medical institution's actual performance, summary can be drawn as follows.

(1) When the value of medical value is defined as the ratio of effect and cost, community hospital B in hypertension diagnosis and treatment has the highest input-output ratio. High-level hospital A takes second place. And C is the last. B, the community hospital, has the best performance in life, health, and social benefits, as well as other vital dimensions of performance, which makes it suitable for clinical such related diseases. Although high-level hospital A is slightly better than community hospital B in all dimensions, it does not have the comparative advantage in the diagnosis and treatment of hypertension for it consumes more medical resources than B. Besides, its economy performance is deficient in the diagnosis and treatment of hypertension, which may lead to the waste of high-quality medical resources. Restricted by medical technology and conditions, mobile diagnosis and treatment platform C has a large gap with hospitals A and B in the dimension of health benefit. However, in the future, treatment platform C can play a more significant role in the field of hypertension diagnosis and treatment utilizing medical association and other means to improve its high level of diagnosis and treatment.

(2) The final results show an increase in the weight of the cause indicators such as the cost of equipment bed and the ratio of doctors to patients and relative reduction in the weight of the result indicators such as patient satisfaction. The result is conducive to finding the root cause that affect the value of medical services and improving the value of medical services systematically.

## Conclusion

This study builds a scientific and comprehensive medical value evaluation system that is better than most of present medical value evaluation. In this study, DEMATEL and information entropy are used to quantify the degree of mutual influence between system indicators and the differences in medical market performance respectively, so as to obtain the objective index weight. This system is a set of objective and accurate evaluation mechanism for the overall multi-attribute value of medical institutions, which can promote the medical institutions for optimizing the utilization efficiency of medical resources and improve their service level as well as patient satisfaction. Besides it is able to enhance the competitive awareness of hospital managers and optimization service awareness.

## Supporting information

S1 Data This file showed the original data and the standardized data in this manuscript. (XLSX)
